# Association of neighborhood level socioeconomic status and patient reported clinical improvement following total shoulder arthroplasty

**DOI:** 10.1016/j.jseint.2024.08.205

**Published:** 2024-09-14

**Authors:** Caleb Morgan, Amanda Firoved, Patrick J. Denard, Justin W. Griffin

**Affiliations:** a Eastern Virginia Medical School, Norfolk, VA, USA; b Marshall University School of Medicine, Department of Orthopaedics, Huntington, WV, USA; c Jordan-Young Institute for Orthopaedic Surgery and Sports Medicine, Virginia Beach, VA, USA; d Oregon Shoulder Institute, Medford, OR, USA

**Keywords:** Socioeconomic, Total shoulder arthroplasty, Neighborhood, Outcome score, SES, Social determinants of health

## Abstract

**Background:**

Prior studies have demonstrated higher preoperative pain and decreased patient-reported outcomes (PROs) following total shoulder arthroplasty (TSA) in individuals with lower socioeconomic status (SES). The goal of this study was to investigate the rate of clinical improvement following TSA in individuals with differing SES.

**Methods:**

Individuals included in this study underwent anatomic or reverse TSA by 2 surgeons between May 2018 and January 2021. Patients were split into 3 SES groups (low, moderate, and high) based on neighborhood SES level as determined by Area Deprivation Index. PROs were collected preoperatively and at 9 weeks, 26 weeks, 1 year, and 2 years postoperatively. Shoulder-specific PROs included the American Shoulder and Elbows Surgeons shoulder score, 10-point visual analog scale for pain, single-assessment numeric evaluation, and Western Ontario Osteoarthritis of the Shoulder Index. The Veterans Rand 12-Item health survey was used to measure overall well-being. We used a mixed-design analysis of variance to determine the interaction of time and improvement in PROs following surgery followed by 1-way mixed-design analysis of variance with post-hoc analysis.

**Results:**

One hundred seventy individuals (low SES n = 34, moderate n = 90, high n = 46) met the inclusion criteria and were included in this study. There were no significant differences between groups for body mass index or age at time of surgery. All groups significantly improved from baseline scores on all PROs (*P* < .001) with the majority of improvement being achieved within the first year after surgery. There were no significant differences in rate of clinical improvement on PROs among the groups when compared to their respective preoperative scores. Significant differences were discovered when comparing groups independent of time with the low- and moderate-SES groups scoring significantly lower on American Shoulder and Elbows Surgeons shoulder score when compared to the high-SES group (*P* < .01) and the low-SES group reporting significantly higher visual analog scale pain when compared to the high-SES group (*P* = .034).

**Conclusion:**

Individuals with lower SES at the neighborhood level report higher pain and decreased shoulder function both preoperatively and postoperatively following TSA; however, the rate of clinical improvement following surgery in this group is comparable to individuals with higher SES when compared to baseline scores. All groups demonstrated significant improvement following surgery, suggesting TSA remains a successful operation regardless of SES.

The importance of social determinants of health (SDOH) in predicting health outcomes and trajectories is becoming increasingly evident as more research and efforts are being made to mitigate its negative effects in disadvantaged populations.^1^,^2^ While there are countless factors that comprise SDOH, it has been defined by the World Health Organization as any condition or circumstance in which people are born, grow, live, work, and age.^11^

A significant component of SDOH is socioeconomic status (SES), which includes components such as income, occupation, education, and housing quality and location. Several studies have demonstrated that individuals experiencing low SES have significantly higher rates of multimorbidity, chronic health conditions, and decreased lifespan.^5^^,^^19^^,^^22^^,^^25^ Studies have also identified higher rates of complications and inferior outcomes following surgical operations in individuals with low SES.^3^^,^^14^^,^^29^

Total shoulder arthroplasty (TSA) is becoming an increasingly common orthopedic procedure to treat a variety of shoulder pathologies including end stage rotator cuff arthropathy (reverse TSA) and osteoarthritis (anatomic TSA).^18^ The procedure is largely considered to be successful and typically yields improved shoulder function and quality of life for the patient.^6^^,^^23^ However, factors shown to negatively impact the success of TSA include the presence of medical comorbidities, psychosocial factors including depression and anxiety, shoulder pathology at time of surgery, preoperative opioid use, and various lifestyle-related behaviors.^9^^,^^16^^,^^21^^,^^30^^,^^34^ Recent studies have also investigated the impact of SES on TSA outcomes due to their known impact on health outcomes.

A recent study conducted by Sheth et al found increased levels of preoperative pain and use of opioids in individuals with lower SES undergoing primary TSA, along with decreased preoperative shoulder function.^26^ Increased rates of preoperative opioid use before TSA in individuals with lower SES were confirmed by another group of authors.^17^ Another study by Waldrop et al found increased preoperative pain and decreased patient-reported outcomes (PROs) in individuals undergoing TSA with Medicare or Medicaid coverage in comparison to those with private insurance.^33^ This study also found worse PROs in the Medicare or Medicaid cohort despite similar objective levels of improvement determined by range of motion measured at a minimum of 2 years postoperatively.

The goal of this study was to further investigate the association between SES at the neighborhood level and PROs at different time points following TSA. We hypothesized that individuals residing in lower-SES neighborhoods will demonstrate lower PROs overall and slower rates of subjective clinical improvement compared to individuals living in high-SES neighborhoods.

## Materials and methods

### Study population

A retrospective study was performed of patient who underwent either anatomic TSA or reverse TSA at 2 institutions between May 2018 and January 2021. Inclusion criteria included primary arthroplasty and a minimum 2-year follow-up. Patients undergoing revision TSA were excluded from this study. Indicated procedures at the time of surgery such as latissimus dorsi transfer, bone grafting, etc. were left to the discretion of the surgeon based on preoperative evaluation and intraoperative findings. Patient demographics such as age, body mass index (BMI), smoking status, and diabetes mellitus (DM) were recorded. This study was reviewed and approved by an Institutional Review Board before commencement (Eastern Virginia Medical School, Norfolk, VA on April 4, 2022; IRB# 22-03-WC-0030).

### Patient-reported outcome measures (PROMs)

Outcomes were collected preoperatively and at 9 weeks, 26 weeks, 1 year, and 2 years postoperatively. All surveys were completed by individuals at scheduled follow-up appointments. Subjective outcome measures included the American Shoulder and Elbow Surgeons shoulder score (ASES), 10-point visual analog scale (VAS) for pain, Western Ontario Osteoarthritis of the Shoulder Index (WOOS), and single-assessment numeric evaluation (SANE). The Veterans Rand 12 (VR-12)–item health survey was used to assess overall physical (physical component score) and mental (mental component score) well-being.

### SES

While there are several different digital tools available to estimate SES, Area Deprivation Index (ADI) was used in this study. ADI has been extensively published in public health–related articles and is a validated measure of SES at the census block–level, the smallest geographic unit for which the United States Census Bureau releases data. It was initially based on a measure created by the Health Resources & Services Administration in the 1990s and was subsequently modified and validated by Amy Kind, MD, PhD, and other researchers at the University of Wisconsin. Lower SES as determined by ADI has been associated with negative health measures such as higher rates of cardiovascular disease, hospital readmission after discharge, increased methylation patterns indicative of aging, decreased performance on executive function tests in individuals at risk for Alzheimer’s, dementia, and others.^13^^,^^15^^,^^27^^,^^35^

**Figure 1:**
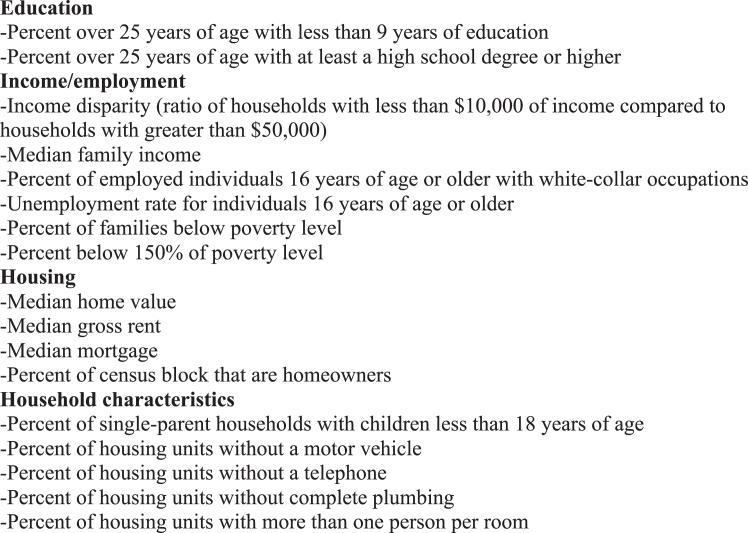
Factors Comprising Area Deprivation Index Scoring in Four Domains: Education, Income/employment, Housing, Household characteristics. ***a*** Adapted from Kind et al.^13^

An individual’s ADI score is determined by the location of their home address within a particular census block and considers 17 different factors in 4 different domains: education, income/employment, housing, and household characteristics (Fig. 1).^13^ These data are collected from the American Community Survey which is distributed by the United States Census Bureau once every 5 years. The score is reported as SES at both the national level (percentile) and state level (decile), with higher percentiles and deciles indicating lower SES.

In this study we used participants’ home addresses associated with their medical record to determine their ADI score. As this study included patients living in different states, we used the national ADI percentile to stratify patients into one of 3 different SES groups: low (percentile>46%), moderate (percentiles 23%-46%), and high (percentiles 0%-22%). These percentile divisions were determined by the difference in ADI percentile from the highest to lowest SES participants and divided by 3.

### Statistical analysis

Mixed-design analysis of variance was used to determine if there was a relationship between SES and clinical improvement at different timepoints as determined by patient-reported outcome measures (PROMs). Post-hoc analysis was performed utilizing Bonferroni analysis. A *P* value < .05 was considered significant. An a priori power analysis was conducted utilizing G∗Power software which revealed a minimum of 27 patients was required for each SES group. All statistical analysis was conducted utilizing IBM SPSS software (version 27.0; IBM Corp., Armonk, NY, USA)

## Results

A total of 170 individuals met the inclusion criteria and were included in this study. There were a total of 34 patients in low-, 90 in moderate-, and 46 in the high-SES group. The mean age at time of surgery was 69.9 (standard deviation = 6.9) and mean BMI was 29.9 (standard deviation = 6.5). There were no significant differences between groups for age (*P* = .229), BMI (*P* = .472), gender (*P* = .205), incidence of DM (*P* = .734) or smoking status (*P* = .943) (Table I).

**Table I:** Sociodemographics of SES groups.

	Low	Moderate	High	Total
N	34	90	46	170
Age	69.37 (7.36)	69.21 (7.07)	71.32 (6.38)	69.89 (6.95)
BMI	29.56 (5.85)	30.45 (6.98)	29.06 (5.09)	29.85 (6.45)
Gender	47.1% female	61.1% female	47.8% female	54.7% female
	52.9% male	38.9% male	52.2% male	45.3% male
Smoker	5.9%	5.5%	4.3%	5.3%
Diabetic	14.7%	12.2%	8.7%	11.8%

*BMI*, body mass index; *SES*, socioeconomic status.

*P > .05* for all metrics as reported in text.

**Figure 2:**
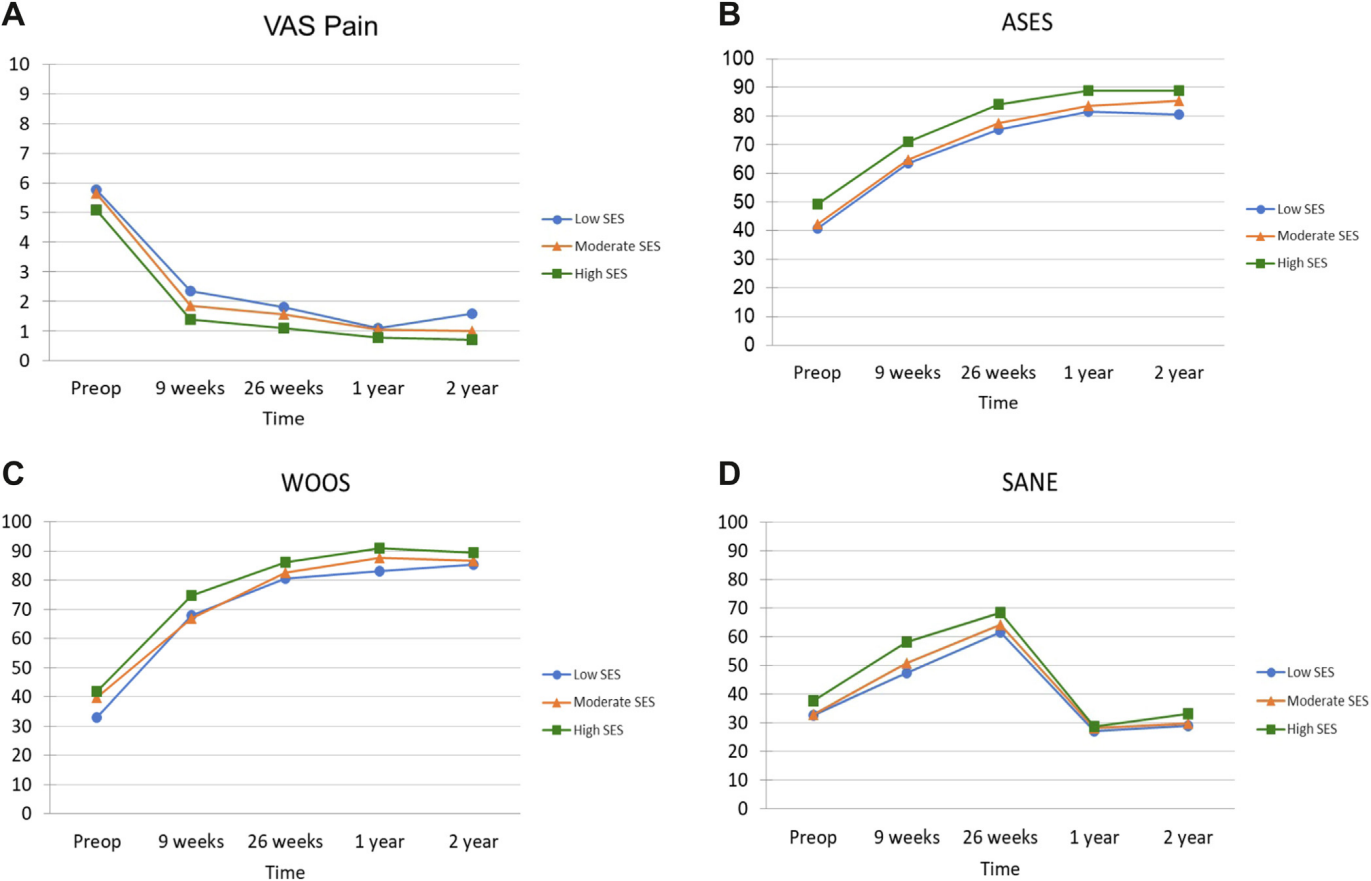
Shoulder PROs collected preoperatively and at time intervals postoperatively following TSA among SES groups. (**A**) Visual analog scale (pain), (**B**) American Shoulder and Elbow Surgeons shoulder score, (**C**) Western Ontario Osteoarthritis of the Shoulder Index, (**D**) single-assessment numeric examination. *PROs*, patient-reported outcomes; *SES*, socioeconomic status; *TSA*, total shoulder arthroplasty; *VAS* pain, pain visual analog scale; *ASES*, American Shoulder and Elbow Surgeons; *WOOS*, Western Ontario Osteoarthritis of the Shoulder Index; *SANE*, single-assessment numeric examination.

**Figure 3:**
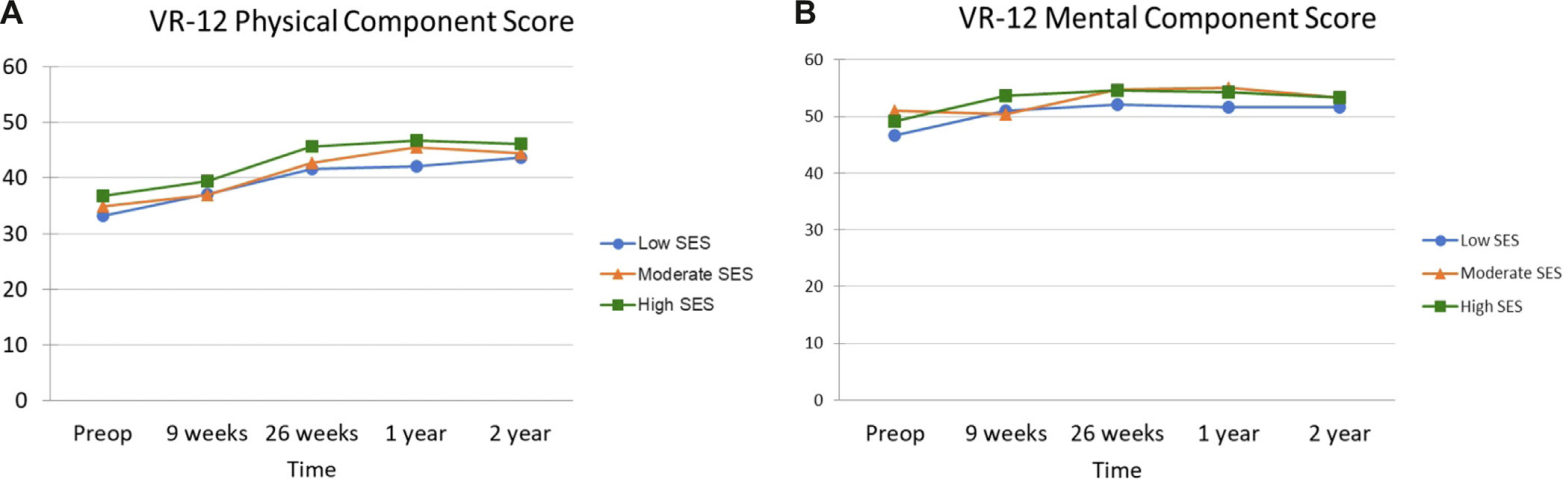
Veterans Rand 12-Item Health Survey collected preoperatively and at postoperative time intervals following TSA among SES groups. (**A**) VR-12 Physical Component, (**B**) Veterans Rand 12-Item Mental Component. *SES*, socioeconomic status; *TSA*, total shoulder arthroplasty; *TSA*, total shoulder arthroplasty; *VR-12*, Veterans Rand 12-item health survey.

In regards to PROMs, there was no association between SES and time, indicating there was no significant difference in rate of clinical improvement between the groups when compared to baseline scores. This is visualized by similar slopes of the graphs seen in Figs. 2 and 3. All groups did significantly improve at the 2-year follow-up for all PROMs when compared to their preoperative baseline scores (*P* < .001; Table II).

**Table II:** Mean (SD) of shoulder PROs collected preoperatively and at postoperative time intervals following TSA among SES groups.

	Low	Moderate	High	Significance
**VAS pain**∗				
Preoperative	5.78 (2.24)	5.638 (1.98)	5.09 (2.25)	*P* < .001
9 weeks	2.35 (2.15)	1.86 (2.07)	1.4 (1.39)	PH; *P* = .034
26 weeks	1.82 (1.92)	1.57 (2.37)	1.11 (1.84)	Low/High
1 y	1.10 (1.52)	1.05 (1.72)	0.78 (1.34)	
2 yrs	1.59 (2.21)	1.00 (1.76)	0.72 (1.27)	
**ASES**∗				
Preoperative	40.83 (16.70)	42.35 (15.83)	49.24 (17.84)	*P < .01*
9 weeks	63.61 (16.96)	64.90 (16.24)	70.96 (14.41)	PH; *P* < .015 Low/High; *P* < .043 Mod/High
26 weeks	75.32 (16.06)	77.70 (20.19)	84.12 (13.61)	
1 y	81.57 (15.62)	83.73 (16.84)	88.84 (13.10)	
2 yrs	80.51 (16.86)	85.35 (16.57)	88.87 (13.30)	
**WOOS**				
Preoperative	32.88 (16.69)	39.81 (21.54)	41.72 (20.53)	*P* = .099
9 weeks	67.80 (20.24)	66.82 (18.97)	74.75 (18.05)	
26 weeks	80.46 (19.41)	82.60 (19.85)	86.24 (15.36)	
1 y	83.17 (19.28)	87.78 (17.79)	90.89 (14.25)	
2 yrs	85.38 (13.95)	86.67 (19.02)	89.36 (16.12)	
**SANE**				
Preoperative	32.66 (23.83)	32.88 (21.23)	37.6 (24.33)	*P* = .150
9 weeks	47.39 (23.80)	50.71 (21.32)	58.09 (21.65)	
26 weeks	61.58 (28.01)	64.29 (28.93)	68.29 (26.85)	
1 y	72.61 (27.18)	71.27 (28.24)	76.73 (28.82)	
2 yrs	67.87 (28.92)	72.65 (29.73)	69.65 (33.13)	

*PROs*, patient-reported outcomes; *SES*, socioeconomic status; *TSA*, total shoulder arthroplasty; *VAS*, Pain (visual analog scale); *ASES*, American Shoulder and Elbow Surgeons shoulder score; *WOOS*, Western Ontario Osteoarthritis of the Shoulder Index; 2d *SANE*, single-assessment numeric examination; *SD*, standard deviation.

PH, Bonforroni post-hoc analysis *P* values.

Low/High = significant post-hoc analysis comparing low to high SES, groups.

Moderate/High = significant post-hoc analysis comparing moderate to high SES, groups.

∗ =Significant, *P* < .05.

In regards to rate of clinical improvement within each group, there was a significant improvement from preoperative scores at each time point up until their 1-year postoperative visit for ASES, WOOS, and SANE (*P* < .001); however, there was no significant difference noted when comparing 1-year PROMs to 2-year PROMs for these measures. Similarly, each group significantly improved in regards to VAS pain (*P* < .001) and VR-12 physical (*P* < .001) at each time point from baseline until 26 weeks postoperatively at which point the groups did not continue to significantly improve. The low-SES group did have an increase in VAS pain between 1 and 2 years postoperatively although it was not significant (Fig. 2*A* and Table II). Furthermore, the high- and low-SES group significantly improved in regard to VR-12 mental scores (*P* < .001) from baseline to 26 weeks postoperatively at which point they did not significantly change (Table III). The moderate-SES group had a decrease in VR-12 mental scores between preoperative and 9-week follow-up although it then significantly increased between the 9- and 26-week follow-up (*P* < .001) (Fig. 3*B*).

**Table III:** Mean (SD) veterans rand-12 item health survey collected preoperatively and at postoperative time intervals following TSA among SES groups.

	Low	Moderate	High	Significance
VR-12 PCS				
Preoperative	33.15 (8.57)	34.90 (8.63)	36.85 (7.61)	*P* = .069
9 weeks	37.09 (9.49)	36.93 (8.17)	39.48 (7.60)	
26 weeks	41.59 (9.80)	42.63 (8.68)	45.68 (8.38)	
1 y	42.13 (10.24)	45.49 (8.61)	46.72 (7.94)	
2 yrs	43.68 (9.58)	44.47 (9.62)	46.09 (7.86)	
VR-12 MCS				
Preoperative	46.58 (12.29)	50.95 (12.56)	49.08 (12.83)	*P* = .365
9 weeks	51.01 (8.05)	50.35 (10.79)	53.63 (10.79)	
26 weeks	52.13 (8.76)	54.82 (10.18)	54.58 (9.22)	
1 y	51.61 (9.75)	55.01 (8.739)	54.35 (10.11)	
2 yrs	51.60 (9.66)	53.35 (10.46)	53.34 (10.92)	

*SES*, socioeconomic status; *TSA*, total shoulder arthroplasty; *VR-12*, Veterans Rand 12-Item; *PCS*, physical component score; *MCS*, mental component score; *SD*, standard deviation.

There were significant differences noted in several PROMs noted between groups when compared to one another independent of time. A significant difference for ASES (*P* < .01) was detected among the groups with post-hoc analysis indicating the high-SES group consistently scored significantly higher when compared to the moderate (*P* = .043) and low-SES groups (*P* = .015) (Table II). Similarly, there was a significant difference between SES groups in regard to VAS pain (*P* = .034) with post-hoc analysis revealing the low-SES group consistently reported significantly higher pain when compared to the high-SES group (*P* = .034). No significant differences were noted between the groups for other measured PROs WOOS (*P* = .099), VR-12 physical (*P* = .069), VR-12 mental (*P = .*365), or SANE (*P* = .150).

## Discussion

The results of our study suggest that lower SES, as determined at the neighborhood level by ADI, is associated with decreased PROs, specifically higher VAS pain and lower ASES scores preoperatively and following TSA. Our results also demonstrate that individuals with differing SES at the neighborhood level appear to clinically improve at similar rates when compared to baseline scores, the majority of which was achieved within the first year following surgery. Furthermore, all groups demonstrated significant improvement in all tested PROs after TSA indicating this procedure is subjectively successful regardless of SES.

Our findings were overall consistent with our hypothesis that individuals with lower SES would report lower PROs. The results are further supported by other publications demonstrating that individuals with lower SES report lower PROs in general in the health-care setting when compared to individuals with higher SES.^20^^,^^32^ While there are several different theories that could help explain our observations, they are most likely multifactorial in nature.

Pain has been extensively studied in different populations and certain associations have been elucidated regarding baseline pain levels. One of these associations is SES and financial insecurity. Individuals with lower SES, in regards to education level, employment, and income level are more likely to suffer from chronic pain than those with higher SES, even when controlling for demographic factors such as race.^10^^,^^24^ Furthermore, studies have also shown that financial insecurity, even when examined on a daily basis, directly relates to pain level.^4^ This association was also found to be true in our study as evidenced by the low-SES groups demonstrating significantly higher VAS shoulder pain both preoperatively and postoperatively when compared to the high-SES group. This is consistent with other publications that have demonstrated low SES having higher preoperative pain levels before TSA.^26^^,^^33^ Preoperative shoulder pain levels have been shown to be a strong predictor of postoperative pain, which is also consistent with the trend of VAS pain seen in the low-SES group in our study.^28^ It should be noted that while there were significant differences in pain between the groups, at 2-year follow-up all groups had relatively low levels of pain.

Shoulder function was also found to be significantly different between SES groups with the low- and moderate-SES groups demonstrating inferior preoperative and postoperative ASES scores. Other studies have also reported similar findings of decreased shoulder function preoperatively in individuals with lower SES.^26^^,^^33^ A possible explanation for these findings is that individuals with lower SES delay treatment with TSA for shoulder dysfunction possibly leading to progressive atrophy of surrounding musculature and advanced arthrosis making recovery after surgery more difficult, although this cannot be proven from our study. Another plausible explanation is that higher pain levels in the lower SES groups negatively impacted scoring on ASES.

Another possible explanation for our findings is that individuals with lower SES are more likely to have medical comorbidities, both physical and psychiatric, which are known to negatively affect health outcomes.^3^^,^^5^^,^^8^^,^^25^^,^^31^ In fact, several recent studies have reported inferior outcomes following TSA for individuals with mood disorders such as anxiety and depression.^7^^,^^30^^,^^34^ While we did not directly control for or report the incidence of mood disorders in our cohort, we did utilize VR-12 mental component score which includes questions regarding depression and anxiety. There were no significant differences between groups for this metric. The association between medical comorbidities such as obesity, diabetes, and tobacco use is also known to negatively affect PROs following TSA.^9^^,^^12^^,^^16^ There were no significant differences noted between the groups for these conditions; however, we did not record or control for other comorbidities such as autoimmune diseases, respiratory conditions, or coronary artery disease. We also used the VR-12 physical component score to assess overall physical health, which revealed no significant differences between the groups.

### Limitations

While our study does provide valuable information regarding the interaction of SES and rate of clinical improvement as determined by PROs following TSA there are numerous limitations. Due to the retrospective cohort design of this study there are inherent limitations such as inaccurate documentation of PROs, sampling error, confounding factors, and confirmation bias. Other limitations include that we did not directly assess for comorbidities outside of obesity, DM, and smoking status. However, we did utilize the VR-12 survey to evaluate general mental and physical health as previously discussed. Furthermore, we used ADI which is a proxy of SES at the neighborhood level rather than an exact measure of SES on an individual basis. Future studies are needed to further elucidate specific potential factors in lower-SES population that lead to decreased shoulder function and higher VAS pain preoperatively and following TSA. Knowing this information would guide future efforts to mitigate these differences.

## Conclusion

Individuals with lower SES at the neighborhood level report higher pain and decreased shoulder function both preoperatively and postoperatively following TSA; however, the rate of clinical improvement following surgery in this group is comparable to individuals with higher SES when compared to baseline scores. All groups demonstrated significant improvement following surgery suggesting TSA remains a successful operation regardless of SES.

## Disclaimers:

Funding: 10.13039/100007307Arthrex Inc provided funding for collection of patient outcomes.

Conflicts of interest: Dr. Patrick J. Denard reports that he is a paid consultant and receives research support and royalties from 10.13039/100007307Arthrex Inc, receives publishing royalties from Wolters Klower 10.13039/100018696Health, and is a paid speaker for Pacira Inc. Dr. Justin W. Griffin reports receiving research support and royalties from 10.13039/100007307Arthrex Inc and is a paid consultant for the same and receives publishing royalties from Springer. The other authors, their immediate families, and any research foundations with which they are affiliated have not received any financial payments or other benefits from any commercial entity related to the subject of this article.

## References

[1] Alegría M., NeMoyer A., Falgàs Bagué I., Wang Y., Alvarez K. (2018). Social determinants of mental health: where we are and where we need to go. Curr Psychiatry Rep.

[2] Allen J., Balfour R., Bell R., Marmot M. (2014). Social determinants of mental health. Int Rev Psychiatry.

[3] Bhattacharyya O., Li Y., Fisher J.L., Tsung A., Eskander M.F., Hamad A. (2021). Low neighborhood socioeconomic status is associated with higher mortality and increased surgery utilization among metastatic breast cancer patients. Breast.

[4] Blyth F.M., van der Windt D., Croft P., Croft P., Blyth F.M., van der Windt D. (2010). Chronic pain epidemiology: From aetiology to public health.

[5] Chetty R., Stepner M., Abraham S., Lin S., Scuderi B., Turner N. (2016). The association between income and life expectancy in the United States, 2001-2014. JAMA.

[6] Cho C.H., Song K.S., Hwang I., Coats-Thomas M.S., Warner J.J.P. (2017). Changes in psychological status and health-related quality of life following total shoulder arthroplasty. J Bone Joint Surg Am.

[7] Colasanti C.A., Lin C.C., Anil U., Simovitch R.W., Virk M.S., Zuckerman J.D. (2023). Impact of mental health on outcomes after total shoulder arthroplasty. J Shoulder Elbow Surg.

[8] Curry E.J., Penvose I., Knapp B., Parisien R.L., Li X. (2021). National disparities in access to physical therapy after rotator cuff repair between patients with Medicaid versus private health insurance. JSES Int.

[9] Iannotti J.P., Norris T.R. (2003). Influence of preoperative factors on outcome of shoulder arthroplasty for glenohumeral osteoarthritis. J Bone Joint Surg Am.

[10] Institute of Medicine (US) Committee on Advancing Pain Research, Care, and Education (2011). https://www.ncbi.nlm.nih.gov/books/NBK92516/.

[11] Islam M.M. (2019). Social determinants of health and related inequalities: Confusion and implications. Front Public Health.

[12] Kamma S.A., Pathapati R.K., Somerson J.S. (2023). Smoking cessation prior to total shoulder arthroplasty: a systematic review of outcomes and complications. Shoulder Elbow.

[13] Kind A.J., Jencks S., Brock J., Yu M., Bartels C., Ehlenbach W. (2014). Neighborhood socioeconomic disadvantage and 30-day rehospitalization: a retrospective cohort study. Ann Intern Med.

[14] LaPar D.J., Bhamidipati C.M., Harris D.A., Kozower B.D., Jones D.R., Kron I.L. (2011). Gender, race, and socioeconomic status affects outcomes after lung cancer resections in the United States. Ann Thorac Surg.

[15] Lawrence K.G., Kresovich J.K., O'Brien K.M., Hoang T.T., Xu Z., Taylor J.A. (2020). Association of neighborhood deprivation with epigenetic aging using 4 clock metrics. JAMA Netw Open.

[16] Mahony G.T., Werner B.C., Chang B., Grawe B.M., Taylor S.A., Craig E.V. (2018). Risk factors for failing to achieve improvement after anatomic total shoulder arthroplasty for glenohumeral osteoarthritis. J Shoulder Elbow Surg.

[17] Morris B.J., Sheth M.M., Laughlin M.S., Elkousy H.A., Edwards T.B. (2020). Risk factors for preoperative opioid Use in patients undergoing primary anatomic total shoulder arthroplasty. Orthopedics.

[18] Norris T.R., Iannotti J.P. (2002). Functional outcome after shoulder arthroplasty for primary osteoarthritis: a multicenter study. J Shoulder Elbow Surg.

[19] Oates G.R., Jackson B.E., Partridge E.E., Singh K.P., Fouad M.N., Bae S. (2017). Sociodemographic patterns of chronic disease: how the mid-south region compares to the rest of the country. Am J Prev Med.

[20] Okunrintemi V., Khera R., Spatz E.S., Salami J.A., Valero-Elizondo J., Warraich H.J. (2019). Association of income disparities with patient-reported healthcare experience. J Gen Intern Med.

[21] Parsons M., Routman H.D., Roche C.P., Friedman R.J. (2019). Patient-reported outcomes of reverse total shoulder arthroplasty: a comparative risk factor analysis of improved versus unimproved cases. JSES Open Access.

[22] Pathirana T.I., Jackson C.A. (2018). Socioeconomic status and multimorbidity: a systematic review and meta-analysis. Aust N Z J Public Health.

[23] Piper C., Neviaser A. (2022). Survivorship of anatomic total shoulder arthroplasty. J Am Acad Orthop Surg.

[24] Portenoy R.K., Ugarte C., Fuller I., Haas G. (2004). Population-based survey of pain in the United States: differences among white, African American, and Hispanic subjects. J Pain.

[25] Shadmi E. (2013). Disparities in multiple chronic conditions within populations. J Comorb.

[26] Sheth M.M., Morris B.J., Laughlin M.S., Elkousy H.A., Edwards T.B. (2020). Lower socioeconomic status is associated with worse preoperative function, pain, and increased opioid use in patients with primary glenohumeral osteoarthritis. J Am Acad Orthop Surg.

[27] Singh G.K., Siahpush M., Azuine R.E., Williams S.D. (2015). Increasing area deprivation and socioeconomic inequalities in heart disease, stroke, and cardiovascular disease mortality among working age populations, United States, 1969-2011. Int J MCH AIDS.

[28] Solberg M.J., Alqueza A.B., Hunt T.J., Higgins L.D. (2017). Predicting 1-year postoperative visual analog scale pain scores and American shoulder and Elbow surgeons function scores in total and reverse total shoulder arthroplasty. Am J Orthop (Belle Mead NJ).

[29] Stamatiou D., Naumann D.N., Foss H., Singhal R., Karandikar S. (2022). Effects of ethnicity and socioeconomic status on surgical outcomes from inflammatory bowel disease. Int J Colorectal Dis.

[30] Vajapey S.P., Cvetanovich G.L., Bishop J.Y., Neviaser A.S. (2020). Psychosocial factors affecting outcomes after shoulder arthroplasty: a systematic review. J Shoulder Elbow Surg.

[31] Valderas J.M., Starfield B., Sibbald B., Salisbury C., Roland M. (2009). Defining comorbidity: implications for understanding health and health services. Ann Fam Med.

[32] von dem Knesebeck O., Bickel H., Fuchs A., Gensichen J., Höfels S., Riedel-Heller S.G. (2015). Social inequalities in patient-reported outcomes among older multimorbid patients – results of the MultiCare cohort study. Int J Equity Health.

[33] Waldrop L.D., King J.J., Mayfield J., Farmer K.W., Struk A.M., Wright T.W. (2018). The effect of lower socioeconomic status insurance on outcomes after primary shoulder arthroplasty. J Shoulder Elbow Surg.

[34] Werner B.C., Wong A.C., Chang B., Craig E.V., Dines D.M., Warren R.F. (2017). Depression and patient-reported outcomes following total shoulder arthroplasty. J Bone Joint Surg Am.

[35] Zuelsdorff M., Larson J.L., Hunt J.F.V., Kim A.J., Koscik R.L., Buckingham W.R. (2020). The area deprivation index: a novel tool for harmonizable risk assessment in Alzheimer's disease research. Alzheimers Dement (N Y).

